# Adolescent Clavicle Fractures: A Management Dilemma?

**DOI:** 10.7759/cureus.77961

**Published:** 2025-01-25

**Authors:** Muhammad Bin A Hamid, Zubair Younis, Muhammad Mannan, Rudra M Prabhu, Nayan Shrivastava, Ali Tauseef, Manjunath A Nagaiah, Ariz Raza, Andalib Kashani

**Affiliations:** 1 Trauma and Orthopaedics, University Hospitals Birmingham National Health Service (NHS) Foundation Trust, Birmingham, GBR; 2 Orthopaedics, The Royal Wolverhampton National Health Service (NHS) Trust, Wolverhampton, GBR

**Keywords:** child and adolescent, clavicle fractures, family medicine, orif, pediatric fractures

## Abstract

Clavicle fractures are among the most common orthopaedic injuries in adolescents, particularly male athletes, arising primarily from sports-related trauma and vehicular accidents. While non-operative treatment remains the standard approach due to favourable recovery outcomes and lower complication rates, the trend toward surgical fixation has gained traction, driven by emerging studies suggesting potential benefits in certain cases. This review critically examines the indications, outcomes, and complications associated with both conservative and operative management of adolescent clavicle fractures. Non-operative treatment demonstrates high healing rates, minimal long-term functional deficits, and excellent patient satisfaction. Conversely, operative interventions, including plate fixation and intramedullary nailing, are associated with improved alignment in displaced fractures but carry risks of hardware-related complications, such as implant irritation, hardware failure, and the necessity for removal surgeries. The role of surgical intervention remains controversial, with no definitive consensus or Level 1 evidence favouring one approach over the other.

## Introduction and background

Clavicle fractures account for some of the most commonly encountered fractures in the adolescent age group [1,2]. In this age group, these fractures are commonly encountered in male athletes, and the mechanism is usually a direct trauma to the affected shoulder. The most common modes of trauma are sports-related injuries, followed by horse-riding, biking, falls and motor vehicular accidents [1,3].

Much has been described regarding non-operative treatment of these fractures in adolescents, with overwhelmingly positive outcomes following this modality of management, and also a relatively faster return to sports following conservative management [4]. Of late, however, there has been an increase in the rate of fixation of clavicle fractures in the adolescent population [5]. This followed on the back of previous studies demonstrating superiority of the same [6,7]. As many studies demonstrate equally good outcomes of non-operative management [8], the complications associated with open reduction and plate fixation must outweigh the benefits associated with surgery for these injuries.

Anatomy

**Figure 1:**
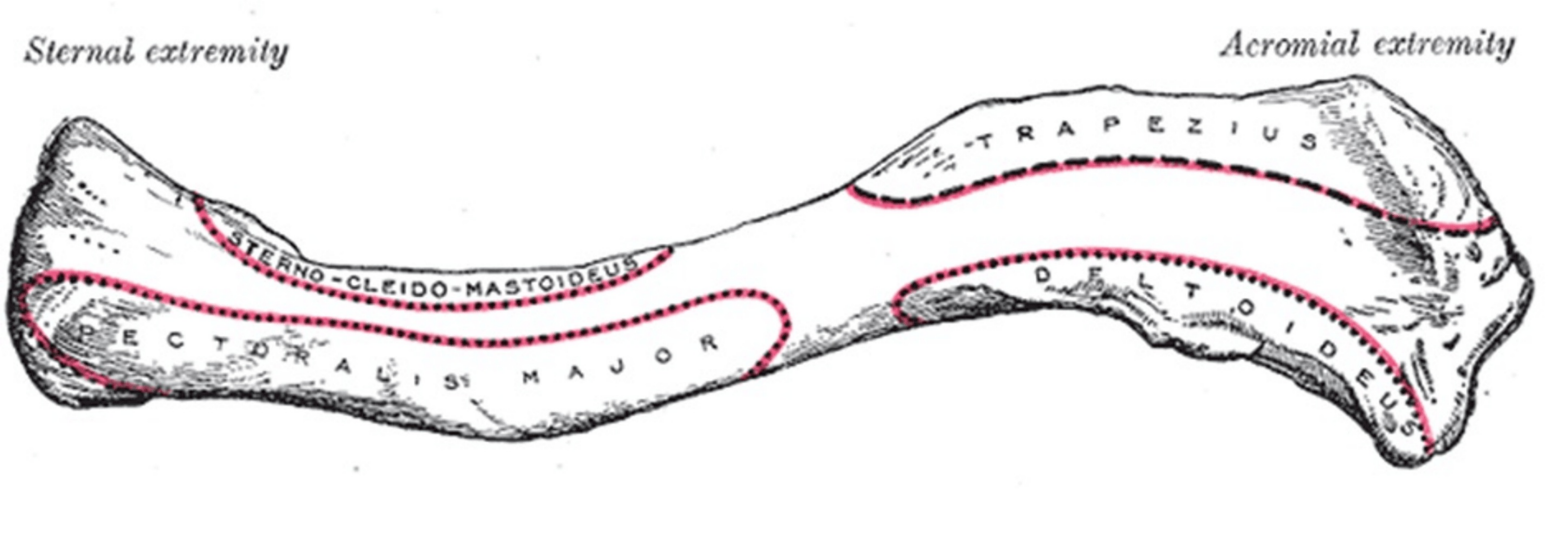
Left clavicle, superior surface. Reproduced with permission from [11].

**Figure 2:**
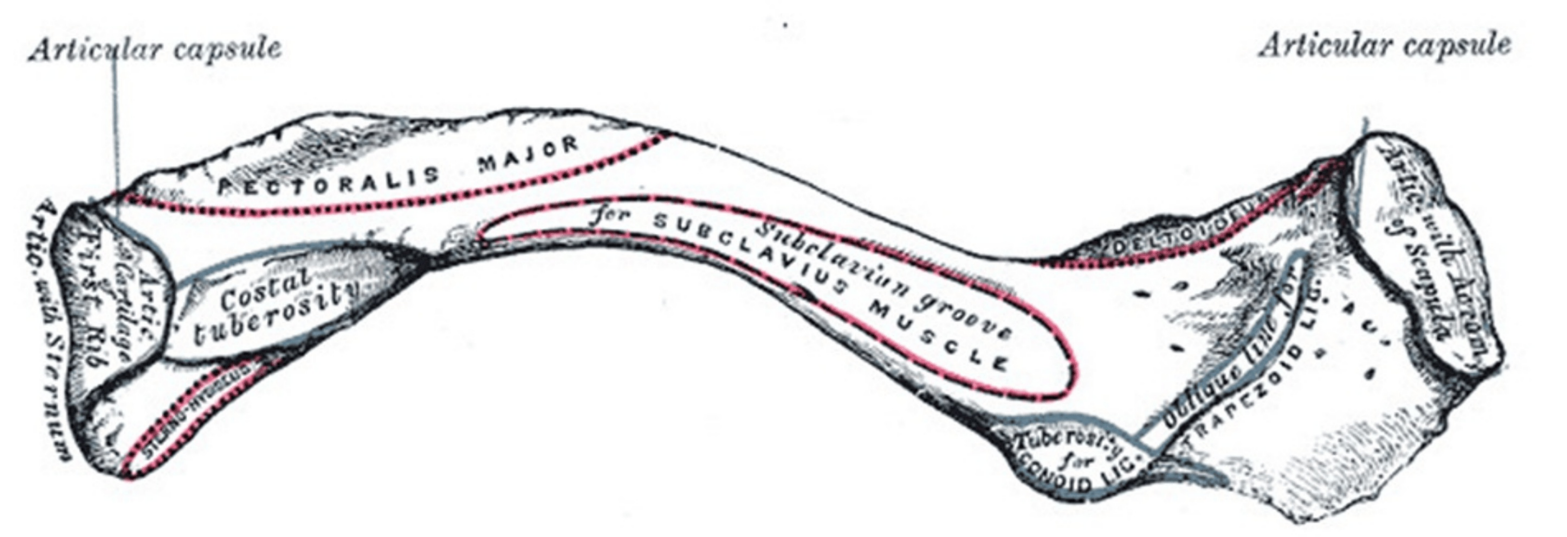
Left clavicle, inferior surface. Reproduced with permission from [11].

The clavicle is the bone connecting the axial to the appendicular skeleton, via the sternoclavicular joint medially and the acromioclavicular joint laterally. Its shape and musculoligamentous attachments serve an important role in providing a platform for a full shoulder range of motion (Figures 1, 2) [9]. It also serves as an important anatomical structure protecting the neurovascular structures that lie in its posterior and inferior relation. Its position also serves to keep the upper extremity at a distance from the thorax, thereby allowing for an unimpeded range of motion of the shoulder [10].

Length, thickness and curvature can vary depending on sex. Males generally have longer, thicker, and more curved clavicle when compared to their female counterparts. A cadaveric study has also shown the left clavicle to be significantly longer than the right [9].

## Review

Clavicle fractures account for 10-15% of all fractures in children [12]. The clavicle commonly fractures due to direct trauma onto the shoulder in 87% of cases. Less commonly, falls on the outstretched hand can also result in a fractured clavicle [13]. Sports-related injuries are the predominant activity leading up to the fracture, with the majority of these fractures being in male individuals [1]. A few decades prior, the rate of surgery in the management of clavicle fractures was a mere 1.6%, whereas today, the rate has been reported to be in the range of 21-26% [14,15].

Non-operative management

The vast majority of clavicle fractures in the paediatric age group are managed conservatively, backed by overwhelming data in support of the same [16]. There is no difference in recovery time in clavicle fractures treated non-operatively: whether using a sling, or a figure-of-eight brace [17]. Midshaft fractures of the clavicle have also been proven to exhibit a much lower non-union rate when compared to similar fractures in adults. The reported rate of non-union in adolescent midshaft clavicle fractures ranges from 0 to 4.8% [18-22]. The same is true for adults, at about 15% [23].

Also, patient-reported outcomes following non-operative treatment have been largely favourable in adolescent clavicle fractures treated non-operatively. Parry et al. conducted a comparison between two small, matched cohorts of adolescents, one with fracture shortening greater than 15 mm and one without, and found no significant differences in outcomes measured by the QuickDASH score and the Constant Shoulder Score [24]. Similarly, Swarup et al. found no differences in patient-reported functional outcomes between operatively treated (43 patients) and nonoperatively treated (36 patients) cohorts, with a median follow-up of 3.8 years [20]. Hagstrom et al. compared 32 nonoperatively treated fractures with 46 operatively treated fractures and found no significant differences in DASH scores, healing times, or time to return to activity [21]. A large prospective study conducted by Heyworth et al. involved 291 adolescents treated nonoperatively [19]. Only two cases (0.7%) of symptomatic malunion were observed. After statistical adjustments were made for confounders, no significant differences in patient-reported outcomes were identified between the treatment groups [19].

**Figure 3:**
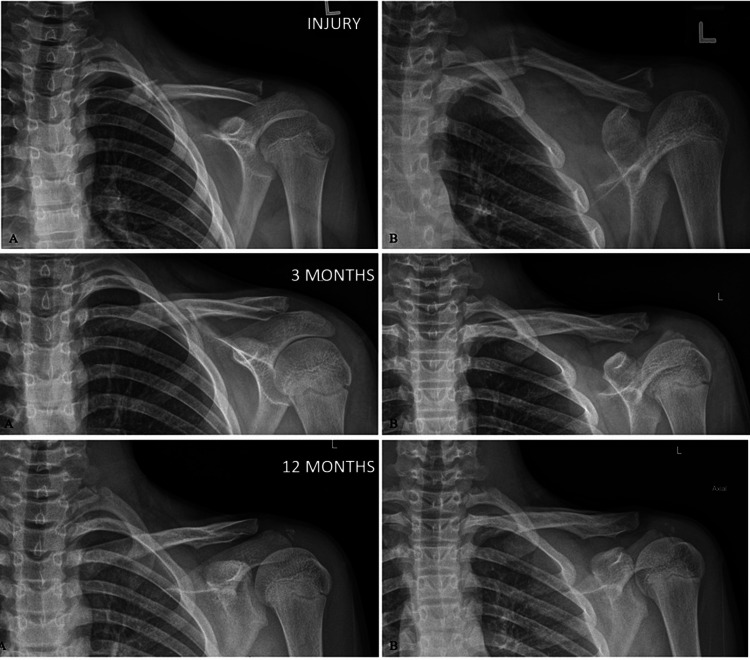
Anteroposterior (A) and Zanca (B) views of the clavicle at injury, three months, and 12 months showing re-modelling potential in a 12-year-old adolescent. Reproduced with permission from [26].

There are studies documenting adverse outcomes following nonoperative treatment. A study by Strauss et al. demonstrated that a completely displaced fracture increased the odds of complications by a factor of 3.2 [25]. Having said that, there are reports that have demonstrated excellent outcomes even in completely displaced segmental fractures (Figure 3) [26].

Operative management

When it comes to operative management, there is considerable evidence among the adult population regarding the fixation of clavicle fractures. There have been studies demonstrating poor outcomes in adults with displaced middle-third clavicle fractures treated conservatively. Some of the problems include persistent pain and reduced strength in the injured extremity [27-29]. Lately, clavicular fractures in adolescents have increasingly been treated with open reduction and internal fixation, more so in the 15 to 19-year-old age group [14]. While the proportion of adolescent clavicle fractures undergoing surgery is rising, there is still no level 1 evidence for or against the same in this age group [30].

Current indications for operative treatment in adolescents include completely displaced midshaft fractures (displacement >2 cm), superior displacement with skin tenting and/or impending compound fractures, fractures with neurovascular injury, open fractures, or floating shoulders with displaced clavicle fractures [30].

By far the commonest modality of fixation for these fractures, and both: superior, and antero-inferior plate placement have been described to yield excellent outcomes in adults [31]. There were initial reports of superior plating being biomechanically superior, but later studies demonstrated similar healing and failure rates with both plate positions, with anteroinferior plate position associated with fewer instances of implant irritation [32]. Dual-plate constructs, while offering better axial load-bearing stiffness over single-plate constructs, have, however, not gained enough traction in this age group [33].

Studies evaluating patient-reported outcomes following clavicle fracture surgery have reported largely favourable results [6,21,34-36]. However, despite good outcomes of osteosynthesis, there have been considerable reports of associated complications that are worth noting [3]. There are numerous reports of complications following either plate fixation, as well as elastic stable intramedullary nailing (ESIN) of the clavicle. The most common complication was a symptomatic implant following clavicle fracture surgery. This often necessitated implant removal. In a retrospective series of 24 children operated on by open reduction and internal fixation, Mehlman et al. removed implants in all patients. However, these removals were elective, and the data on symptomatic implants was limited in their report [6]. In a report by Vander Have et al., 18% of patients who were operated by plate fixation had their implants removed owing to symptomatic metalwork [7]. In the series by Namdari et al., 29% of children underwent implant removal after plate fixation of clavicle fractures [34]. Randsborg et al. operated on nine adolescent clavicle fractures and had to perform implant removal surgery on six patients because of local irritation [37]. In yet another study, implant-related symptoms were seen in 13% of children, and the rate of metalwork removal was 9% in operatively treated midshaft clavicle fractures in adolescents [38].

ESIN and K-wires have both been described to achieve intra-medullary fixation in clavicle fractures. They are not the best modality of fixation when there is comminution, as they tend to suffer from telescoping and subsequent non-union [39]. Also, intramedullary fixation methods are technically demanding and have higher rates of hardware failure/irritation, coupled with prolonged surgery times [7,40]. In a study by Rapp et al., among the 24 children operated by ESIN, one suffered nail breakage, two had nail bending necessitating revision, and two had nails causing impending skin penetration [35]. In another study, out of four nails, one failed and had to be revised to plate fixation, and one nail led to a re-fracture of the clavicle. In the same study, out of the 19 patients operated using plate fixation, four had complications. One had refracture, two implant removals and one non-union with implant failure [3].

In a paper by Kim et al, out of the 70 adolescents treated by surgery, there were nine patients with metal-induced irritation [41]. Out of these nine, four were fixed using ORIF (open reduction and internal fixation) with plate and screws, three using MIPO (minimally invasive plate osteosynthesis) plating, and two using threaded Steinmann pins [41].

Another factor often brought up during the decision-making process is cosmesis. While shortened, displaced fractures benefit from operative restoration of alignment; they also come with a resultant scar following surgery. At the same time, conservative management may often leave undesirable cosmesis in the form of visible bumps (callus), or residual deformity. In studies that have assessed satisfaction, there are similar rates of dissatisfaction in non-operative, as well as operatively treated cohorts in adolescent clavicle fractures [15,37].

## Conclusions

Given the above due consideration, it is still a matter of considerable debate on what is the optimal method of treatment for adolescent clavicle fractures. This highlights the importance of individualized treatment plans considering patient age, fracture characteristics, cosmesis, and growth potential, to optimize outcomes in adolescent clavicle fractures. Patients and their parents must be involved in the decision-making process and enabled to make an informed choice based on their clinical picture and fracture characteristics. Further high-quality studies are needed to establish clear management guidelines for this population. There are many confounding factors in this regard, and none of the prior studies have helped create a treatment algorithm for the management of these injuries. Remodelling potential decreases with an increase in age, and any study that aims to evaluate this must take into account years of growth remaining as a marker of possible remodelling potential before arriving at a conclusion.
